# Medikamenteninteraktion zwischen Ceftriaxon und Theodrenalin/Cafedrin: Ein Fallbeispiel

**DOI:** 10.1007/s00101-022-01217-5

**Published:** 2022-11-21

**Authors:** Christina Werzer, Simon Schäfer, Klaus Hofmann-Kiefer

**Affiliations:** Klinik für Anaesthesiologie am Klinikum der Universität München, Ludwig-Maximilians-Universität München, Campus Innenstadt, Nussbaumstraße 20, 80336 München, Deutschland

**Keywords:** Medikamenteninteraktion, Theodrenalin/Cafedrin, Ceftriaxon, Perioperative Antibiotikaprophylaxe, Intraoperative Hypotonie, Drug interaction, Theodrenaline/cafedrine, Ceftriaxone, Perioperative antibiotic prophylaxis, Intraoperative hypotension

## Abstract

Intravenöse Medikamenteninteraktionen finden im Bereich der Intensivmedizin immer größere Beachtung, da dort eine Vielzahl von Medikamenten über eine begrenzte Anzahl an i.v.-Zugängen appliziert werden muss; doch treten sie auch im Routinebetrieb im OP auf. Der folgende Fallbericht soll die Aufmerksamkeit auf die Medikamentenkombination Theodrenalin/Cafedrin mit verschiedenen Antibiotika lenken.

## Abstract (en)

Adverse interactions between intravenous medications which are given simultaneously are a common problem in intensive care medicine. They are usually caused by administering a high number of medications over a limited number of intravenous lines or central venous catheters; however, this issue also arises in routine anesthetic procedures during surgery. The following case report highlights a so far undocumented interaction between the combination of theodrenaline/cafedrine and various antibiotics.

Laparoscopic cholecystectomy was performed in a female patient, classified as ASA 1. After induction of general anesthesia 2 g ceftriaxone were administered as a perioperative antibiotic prophylaxis. Simultaneously, i.e. prior to the beginning of surgery, a mild decrease in blood pressure was observed and 2 ml diluted Akrinor® (2 ml theodrenaline/cafedrine + 8 ml NaCl 0.9%) was administered. Directly following this administration a chemical precipitation reaction occurred, and large white pasty flakes were noticed in the intravenous line. The infusion was stopped immediately and all lines were replaced.

In order to confirm a causal relationship between the observed precipitation and the simultaneous administration of the two drugs, an in vitro test was performed by mixing Akrinor® with other preparations of cephalosporin antibiotics. The effect observed with ceftriaxone was reproducible and cefazoline also caused a precipitation reaction.

## Anamnese

Bei einer circa 30- bis 35-jährigen, kardiopulmonal gesunden Patientin (158 cm, 58 kg) wurde im Rahmen einer symptomatischen Cholezystolithiasis eine laparoskopische Cholezystektomie in Allgemeinanästhesie durchgeführt. Bekannte Vornarkosen waren anamnestisch problemlos. Es wurde ein 20-Gauge-Zugang am linken Handrücken gelegt und die Narkose mit 20 µg Sufentanil, 150 mg Propofol (fraktioniert 100; 30; 20 mg) und 6 mg Cisatracurium eingeleitet. Die Narkoseaufrechterhaltung erfolgte mittels Sevofluran. Im Rahmen der Lagerung kam es zu einer kurzfristigen Hypotension (mittlerer arterieller Blutdruck: 59 mm Hg), woraufhin 2 ml einer verdünnten Theodrenalin/Cafedrin-Mischung (2 ml Theodrenalin/Cafedrin + 8 ml 0,9 %ige NaCl-Lösung) verabreicht wurden. 2 ml Theodrenalin/Cafedrin, Handelsname Akrinor®^,^ (Fa. Ratiopharm, Ulm, Deutschland) enthalten 10 mg Theodrenalinhydrochlorid und 200 mg Cafedrinhydrochlorid (Verhältnis 1:20) [1].

**Figure 1:**
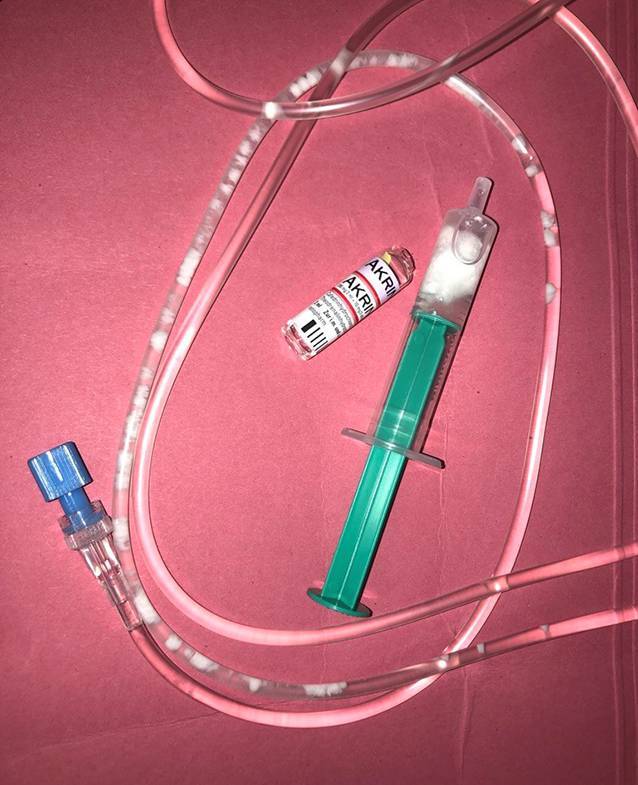

Der linke Arm wurde angelagert, die Infusionsleitung mittels Heidelberger-Verlängerung verlängert. Bei gutem Venenstatus wurde primär auf einen zweiten i.v.-Zugang verzichtet. Nach dem Team-Time-Out wurde eine prophylaktische Single-Shot-Gabe von 2 g Ceftriaxon in 100 ml Aqua ad iniectabilia verabreicht; die Jonosteril®-Grundinfusion wurde in der Zwischenzeit gestoppt. Unmittelbar vor dem Schnitt – noch bei laufender Ceftriaxon-Infusion – kam es erneut zu einer milden Hypotension, bei der wiederum 2 ml der Akrinor®-Mischung, diesmal über die Zuspritzmöglichkeit der Heidelberger-Verlängerung, gegeben wurden. Sofort kam es zu einer Ausfällreaktion im Schlauchsystem, bei der sich dicke, weiße Flocken bildeten (Abb. 1). Die Ceftriaxon-Infusion wurde gestoppt, noch bevor die Präzipitate das Gefäßsystem der Patientin erreichen konnten. Die Infusionsschläuche wurden gewechselt und ein zweiter i.v.-Zugang gelegt. Nun wurden Antibiose und Akrinor® mit Jonosteril® (Fa. Fresenius Kabi Deutschland GmbH, Bad Homburg, Deutschland) als Trägerlösung getrennt gegeben. Die Patientin war zu keiner Zeit vital bedroht; durch das rasche Handeln konnte die Aufnahme des Präzipitats in das Venensystem der Patientin verhindert werden.

## Verlauf

**Figure 2:**
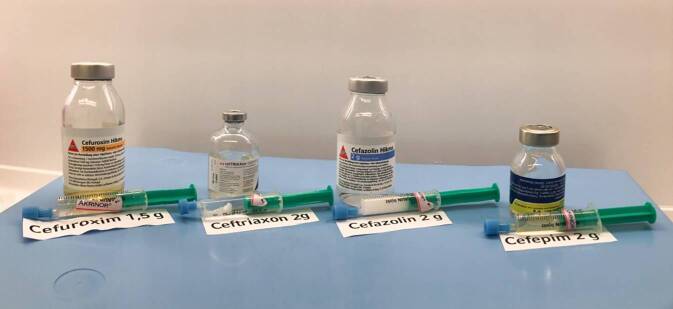

Der Verdacht lag nahe, dass die Ausfällreaktion durch die Vermischung von Ceftriaxon und Akrinor® ausgelöst wurde. Nach beendeter Ceftriaxon-Infusion wurden zur Ursachenforschung einige Milliliter aus dem Infusionsschlauch abgezogen und mit Akrinor® in einer Spritze gemischt. Sofort darauf bildete sich wieder ein weißes, klumpiges Präzipitat (Abb. 2). Beim Versuch, die Spritze zu leeren, blieb ein fester, lehmiger Überstand in der Spritze zurück. Der Fall wurde dokumentiert und als Critical-Incident-Fall der Klinikapotheke gemeldet.

## Literaturrecherche

Eine Literaturrecherche in den Datenbanken Embase und Pub-Med ergab keine weiteren Fallberichte über die Inkompatibilität von Akrinor® und Ceftriaxon, wobei die englischsprachige Literatur kaum Untersuchungen zu Akrinor® enthält. Fast alle Publikationen zu Akrinor® stammen aus dem deutschsprachigen Raum.

## Eigene Untersuchungen

Um mögliche weitere Ausfällreaktionen bei anderen Cephalosporinen mit Akrinor® zu detektieren, wurde in einem kleinen Versuchsmodell untersucht, wie sich die Kombination mit 4 weiteren Cephalosporinen verhält.

**Figure 3:**
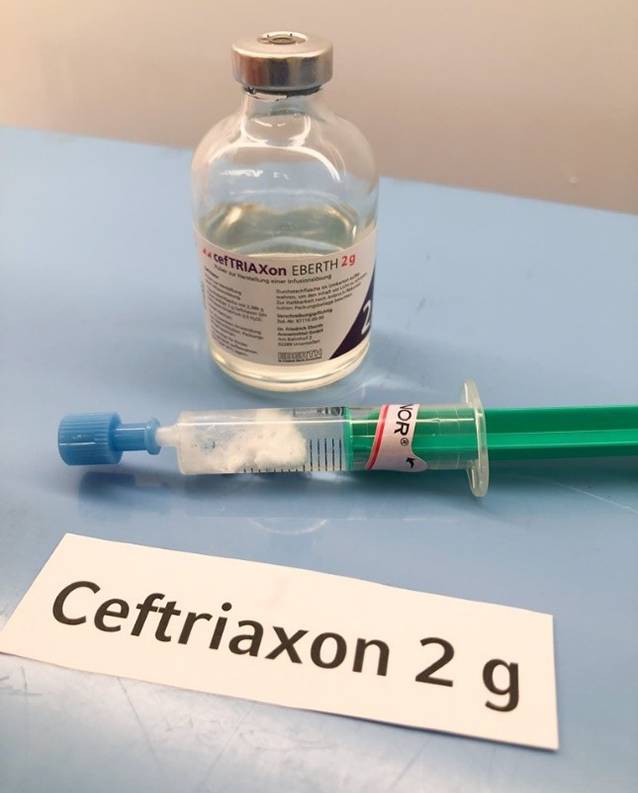

**Figure 4:**
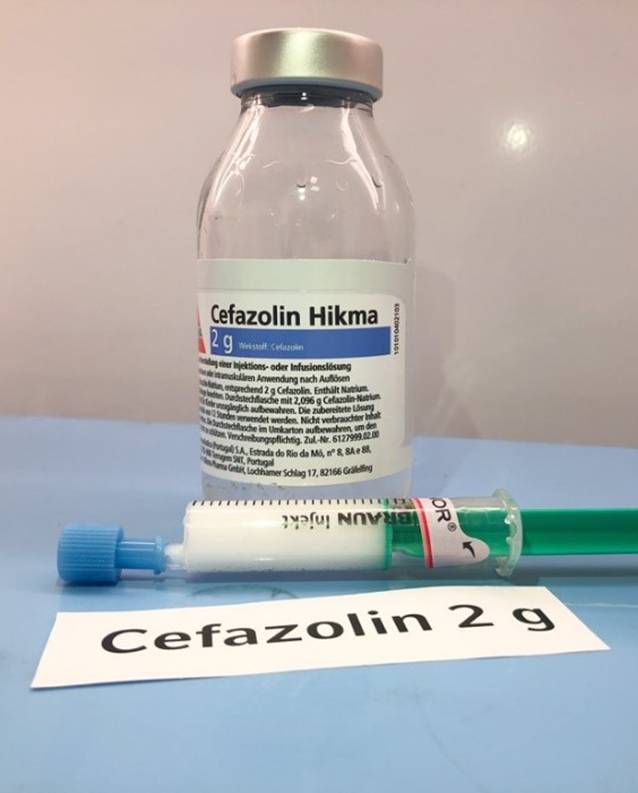

**Figure 5:**
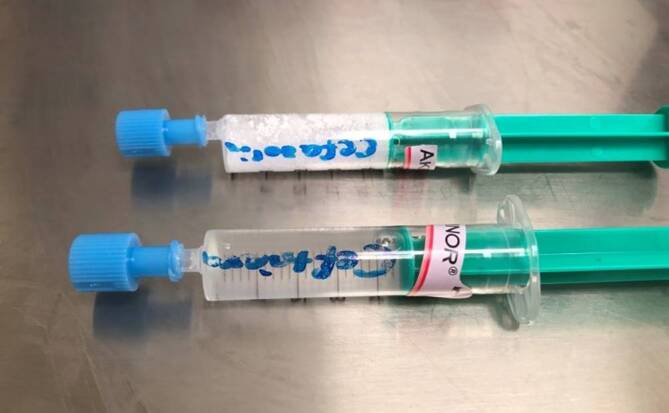

Dazu wurden Cefuroxim 1,5 g (Hikma Farmacêutica, Portugal S.A.), Cefazolin 2 g (Hikma Farmacêutica, Portugal S.A.), Cefepim 2 g (MiP Pharma Holding GmbH, Deutschland) und zur Kontrolle nochmals Ceftriaxon gemäß den Vorgaben der Fachinformationen in Aqua ad iniectabilia gelöst. Die 2 ml der Akrinor®-Ampulle wurden pur in 5‑ml-Spritzen aufgezogen. Mit einer frischen Kanüle wurden nun jeweils 2–3 ml der Antibiotikalösung in jeweils eine vorbereitete Akrinor®-Spritze aufgezogen und die Reaktion beobachtet. Bei Ceftriaxon ließ sich die grobflockige Ausfällung reproduzieren (Abb. 3); bei Cefazolin färbte sich die Mischung milchig weiß (Abb. 4). Keine makroskopische Reaktion konnte bei Cefuroxim und Cefepim beobachtet werden. Nach 2 h konnte man ein Auflösen der Ceftriaxon-„Kristalle“ beobachten; die erst milchig-trübe Cefazolin-Mischung flockte über den Zeitraum zunehmend aus (Abb. 5). Diese Beobachtungen stellen lediglich Stichproben dar und erheben keinen Anspruch auf Vollständigkeit!

## Diskussion

Akrinor® ist seit 1963 in Deutschland zugelassen und hier ein beliebtes Medikament zur Therapie der perioperativen Hypotonie mit Wirkung vorrangig an den β‑Rezeptoren. Neben Theodrenalin und Cafedrin enthält die Präparation noch den Stabilisator Natriummetabisulfit (max. 0,4 mg entspr. max. 0,27 mg SO_2_/Amp.) sowie Ethanol 200 mg/Amp., Glycerol 85 %, Natriumacetat-Trihydrat und 99 %ige Essigsäure und Wasser für Injektionszwecke. Laut Fachinformation enthält 1 Amp. Akrinor® zudem 12 Vol.-% Alkohol [1, 2]. Genauere pharmakologische Untersuchungen bezüglich Plasmaverteilung und Plasmahalbwertszeit (laut Fachinformation 1 h) gibt es vorrangig für Cafedrin; zu Theodrenalin gibt es kaum verwertbare Informationen [3].

Ceftriaxon ist ein Gruppe-3a-Cephalosporin. Es hat eine Halbwertszeit von 7–8 h [4]. Ceftriaxon 2 g Eberth, Dr. Friedrich Eberth Arzneimittel GmbH, 92289 Ursensollen, Deutschland, (das in diesem Fall verwendet wurde) wird als Trockensubstanz mit dazugehörigem Lösungsmittel (40 ml 0,9 %ige NaCl-Lösung) geliefert. 2,386 g Pulver enthalten 2 g Ceftriaxon (als Ceftriaxon-Dinatrium 3,5 H_2_O). Der pH-Wert der gebrauchsfertigen Lösung liegt bei 6–8. Laut Literatur ist Ceftriaxon (nur) mit Fluconazol und Aminoglykosiden physikalisch nicht kompatibel [5, 6].

Im Gegensatz zu dem aktuellen Fallbericht vielfach beschrieben ist eine Ausfällung von Ceftriaxon in Anwesenheit von Kalzium, die v. a. in der Neonatologie von Bedeutung ist [7]. Bereits um die Jahrtausendwende kam es zu Berichten über eine gehäufte Mortalität bei Kindern unter einem Jahr, welche Ceftriaxon und Kalzium über das gleiche Infusionssystem erhalten hatten. Die Präzipitate hatten sich in den kleinen Gefäßen, v. a. in der Lungenstrombahn, angesammelt und zu Embolien geführt. Seit 2007 ist nach Forderung der Food and Drug Administration (FDA) der Fachinformation ein entsprechender Hinweis zu entnehmen [8, 9]. Kalzium ist u. a. auch in den gängigen Infusionslösungen Jonosteril® und Ringer-Lactat-Lösung (z. B. Ringer-Lactat-Lösung nach Hartmann B. Braun) enthalten [10]. In der Fachinformation von Ceftriaxon wird ausdrücklich darauf hingewiesen, dass Ringer-Lactat-Lösung kein geeignetes Lösungs- oder Verdünnungsmittel für Ceftriaxon darstellt [8]. Im aktuellen Fall wurde das kalziumhaltige Jonosteril® aber nicht gleichzeitig mit Ceftriaxon verabreicht.

Chemisch betrachtet kommen außer den oben angesprochenen Mechanismen noch folgende Ursachen für eine Ausfällreaktion infrage: Wird das Löslichkeitsprodukt einer Substanz in einer Lösung überschritten, kommt es zu einer Übersättigung, chemisch als Fällungsreaktion bezeichnet, und es bildet sich ein Niederschlag. Diese Ursache erscheint jedoch im oben genannten Beispiel als unwahrscheinlich, denn es lassen sich bis zu 40 g Ceftriaxon in 100 ml Wasser lösen, weitaus mehr als für den klinischen Gebrauch notwendig [5, 11]. Weitere Ursachen können eine ionische Wechselwirkung der 2 gemischten Substanzen bei unterschiedlicher Molekülladung, aber auch Temperaturschwankungen oder eine Verschiebung des pH-Werts sein [12]. Da Temperaturschwankungen in unserem Fall nicht auftraten, kommen am ehesten eine Verschiebung des pH-Werts oder eine ionische Wechselwirkung als Ursache der Präzipitatbildung in Betracht.

Die gebrauchsfertige Ceftriaxon-Lösung ist weitgehend pH-neutral (pH 6–8). Der Akrinor®-Lösung ist sowohl Na-Hydroxid als auch Na-Acetat und Essigsäure (Na-Acetat/Essigsäure-Puffer) zur pH-Wert Stabilisierung beigefügt, was auf eine eher alkalische Ausgangslage der weiteren Inhaltsstoffe schließen lässt. In der Literatur finden sich hierzu allerdings keine Angaben. Beide Medikamente sind formal ungeladen. Letztlich lässt sich daher auf einfachen Wegen keine der möglichen Ursachen der Ausfällreaktion nachweisen oder zumindest als wahrscheinlich einstufen. In der Literatur wurden keine Angaben zu möglichen Wechselwirkungen zwischen Ceftriaxon und den in Akrinor® enthaltenen Zusatzstoffen gefunden. Grundlegendere pharmakologische Untersuchungen, als sie im Rahmen dieses Fallbeispiels möglich sind, müssten durchgeführt werden, um den Ursachen der Ausfällreaktion „auf die Spur zu kommen“. Dieser Artikel soll daher vornehmlich dazu dienen, andere Anwender auf das Problem hinzuweisen und davor zu warnen, Akrinor® und Ceftriaxon über denselben Infusionsschenkel zu verabreichen.

## Fazit für die Praxis

- Bei der zeitgleichen Infusion von Akrinor® und Ceftriaxon über einen Infusionsschenkel kam es zu einer schweren Ausfällreaktion; es konnte kein pharmakologisch plausibler Zusammenhang gefunden werden.
- Im OP-Betrieb sollte explizit auf die Auswahl der Lösungsmedien von parallel laufenden Infusionen und Medikamenten sowie auf mögliche bekannte Inkompatibilitäten geachtet werden
- Ist die Gabe von Akrinor® in eine laufende Ceftriaxon-Lösung unvermeidbar (z. B. sofortige Kreislaufstabilisierung), sollte der patientennahe Infusionsschenkel vor und nach der Injektion mit ausreichend 0,9 %iger NaCl-Lösung gespült werden.
